# Diagnostic differences in respiratory breathing patterns and work of breathing indices in children with Duchenne muscular dystrophy

**DOI:** 10.1371/journal.pone.0226980

**Published:** 2020-01-10

**Authors:** Lauren Ryan, Tariq Rahman, Abigail Strang, Robert Heinle, Thomas H. Shaffer

**Affiliations:** 1 Department of Biomedical Research, Nemours/Alfred I. duPont Hospital for Children, Wilmington, Delaware, United States of America; 2 Division of Pulmonary Medicine, Nemours/Alfred I. duPont Hospital for Children, Wilmington, Delaware, United States of America; 3 Center for Pediatric Lung Research, Nemours/Alfred I. duPont Hospital for Children, Wilmington, Delaware, United States of America; Cleveland Clinic, UNITED STATES

## Abstract

**Rationale:**

Pulmonary function testing (PFT) provides diagnostic information regarding respiratory physiology. However, many forms of PFT are time-intensive and require patient cooperation. Respiratory inductance plethysmography (RIP) provides thoracoabdominal asynchrony (TAA) and work of breathing (WOB) data. *pneu*RIP^TM^ is a noninvasive, wireless analyzer that provides real-time assessment of RIP via an iPad. In this study, we show that *pneu*RIP^TM^ can be used in a hospital clinic setting to differentiate WOB indices and breathing patterns in children with DMD as compared to age-matched healthy subjects.

**Methods:**

RIP using the *pneu*RIP^TM^ was conducted on 9 healthy volunteers and 7 DMD participants (ages 5–18) recruited from the neuromuscular clinic, under normal resting conditions over 3–5 min during routine outpatient visits. The tests were completed in less than 10 minutes and did not add excessive time to the clinic visit. Variables recorded included labored-breathing index (LBI), phase angle (Φ) between abdomen and rib cage, respiratory rate (RR), percentage of rib cage input (RC%), and heart rate (HR). The data were displayed in histogram plots to identify distribution patterns within the normal ranges. The percentages of data within the ranges (0≤ Φ ≤30 deg.; median RC %±10%; median RR±5%; 1≤LBI≤1.1) were compared. Unpaired t-tests determined significance of the data between groups.

**Results:**

100% patient compliance demonstrates the feasibility of such testing in clinical settings. DMD patients showed a significant elevation in Φ, LBI, and HR averages (*P*<0.006, *P*<0.002, *P*<0.046, respectively). Healthy subjects and DMD patients had similar BPM and RC% averages. All DMD data distributions were statistically different from healthy subjects based on analysis of histograms. The DMD patients showed significantly less data within the normal ranges, with only 49.7% Φ, 48.0% RC%, 69.2% RR, and 50.7% LBI.

**Conclusion:**

In this study, noninvasive *pneu*RIP^TM^ testing provided instantaneous PFT diagnostic results. As compared to healthy subjects, patients with DMD showed abnormal results with increased markers of TAA, WOB indices, and different breathing patterns. These results are similar to previous studies evaluating RIP in preterm infants. Further studies are needed to compare these results to other pulmonary testing methods. The *pneu*RIP^TM^ testing approach provides immediate diagnostic information in outpatient settings.

## Introduction

Duchenne muscular dystrophy (DMD) is a disabling genetic disorder, due to an X-linked myopathy that prevents the production of dystrophin, a normal muscle protein. Because of the recessive genetic mutation, DMD affects mostly boys. However, the severity of muscular dystrophies is associated with pathogenic variants in DMD, which encodes the protein dystrophin. The phenotype can range from mild to severe, depending on age and disease progression. Most patients with severe DMD die in their second to third decade from muscle-associated respiratory and cardiovascular complications [1,2].

Currently, there is no cure for DMD; however, there are promising treatments directed at symptoms such as scoliosis [3] and respiratory muscle weakness (and overuse of steroids) [2,4]. Cough-assisted devices for pulmonary hygiene as well as pharmaceutical and gene-replacement therapies [5] are also available, extending and improving the quality of life.

As a pediatric disorder, DMD clinically presents at around age two years and results in progressive respiratory muscle weakness [1]. Eventually, these patients experience lung dysfunction that often leads to chronic respiratory failure and may require long-term ventilatory support. Therefore, assessment and management of pulmonary function in the DMD population is essential to the survival of DMD patients [2,5] and should be routinely performed for every DMD patient, at least at diagnosis and during follow-up, since interventional therapy needs to start early in the disease process [1,6]. In this regard, several DMD pediatric studies have evaluated various pulmonary function testing (PFT) paradigms for respiratory function analysis in children with neuromuscular disease [7]. Regular use of these tests is recommended for lung function assessment; however, many forms of PFT are time-intensive and require patient cooperation, so the feasibility of these tests for providing real-time data in numerous pediatric clinical settings is limited [8]. Respiratory inductance plethysmography (RIP) provides thoracoabdominal asynchrony (TAA) and work of breathing (WOB) index data, as well as patterns of breathing. The RIP technique has been shown in multiple studies to noninvasively provide useful diagnostic results in pediatric patients with a variety of respiratory disorders, including asthma, neonatal lung disease, and neuromuscular (NM) disease [9–12]. The *pneu*RIP^TM^, a newly developed research tool, is a noninvasive, wireless analyzer that provides real-time assessment of RIP via an iPad. RIP requires minimal patient cooperation and can be performed without adding excessive time to the clinic visit.

In this study, we hypothesize that pneuRIP^TM^ can demonstrate differential WOB indices and breathing patterns in children with DMD as compared to age-matched healthy subjects in a hospital clinic setting. Furthermore, we speculate that the *pneu*RIP^TM^ testing approach may provide useful information in both inpatient and outpatient settings and possibly in-home settings, since information can be transmitted securely to hospital records. From a clinical perspective, the new mutation- and gene-specific therapies for NM diseases, like DMD, are likely to confer long-term therapeutic benefit. Thus, appropriate, noninvasive approaches to assess respiratory function will be required to follow therapeutic interventions in children with DMD, particularly the youngest patients who cannot perform standard pulmonary testing. In this feasibility study, we show that *pneu*RIP^TM^, can be used to assess lung function and note differences between children with DMD and healthy subjects.

## Methods

This study was approved by the Nemours institutional review board (IRB: Study# 613308), and all participants gave written consent/assent. As such, written informed consent/assent was sought from the patients or their parents/guardians (for patients younger than 18 years). All 7 patients were diagnosed genetically and clinically with DMD by our multidisciplinary clinical team (orthopedics, neurology, genetics, pulmonology, nutrition). Comorbidities in the DMD study group included obesity (2 patients), obstructive sleep apnea (2 patients), cardiomyopathy (1 patient), and wheelchair use in 5 of the 7 patients (Table 1).

**Table 1:** Characteristics of study groups.

Data of Study Groups		Healthy		Patients	
**Total**		9		7	
**Sex**	M	9 (100%)		7 (100%)	
	F	0 (0%)		0 (0%)	
**Age (yr)** Mean ± SD		12.7 (±5.2)		12.2 (±4.7)	
**DMD Patients**					
**Subject #**		**Age (yr)**	**Race**	**Co-Morbidities**	**Wheelchair users**
1		7	C	Snoring	no
2		17	C	OSA, Cardio	yes
3		13.5	C	OSA, Obesity	yes
4		18	C	None	yes
5		11.5	C	None	yes
6		14	C	Obesity	yes
7		5	C	None	no

OSA = Obstructive sleep apnea; Cardio = cardiomyopathy; C = Caucasian

**Fig 1:**
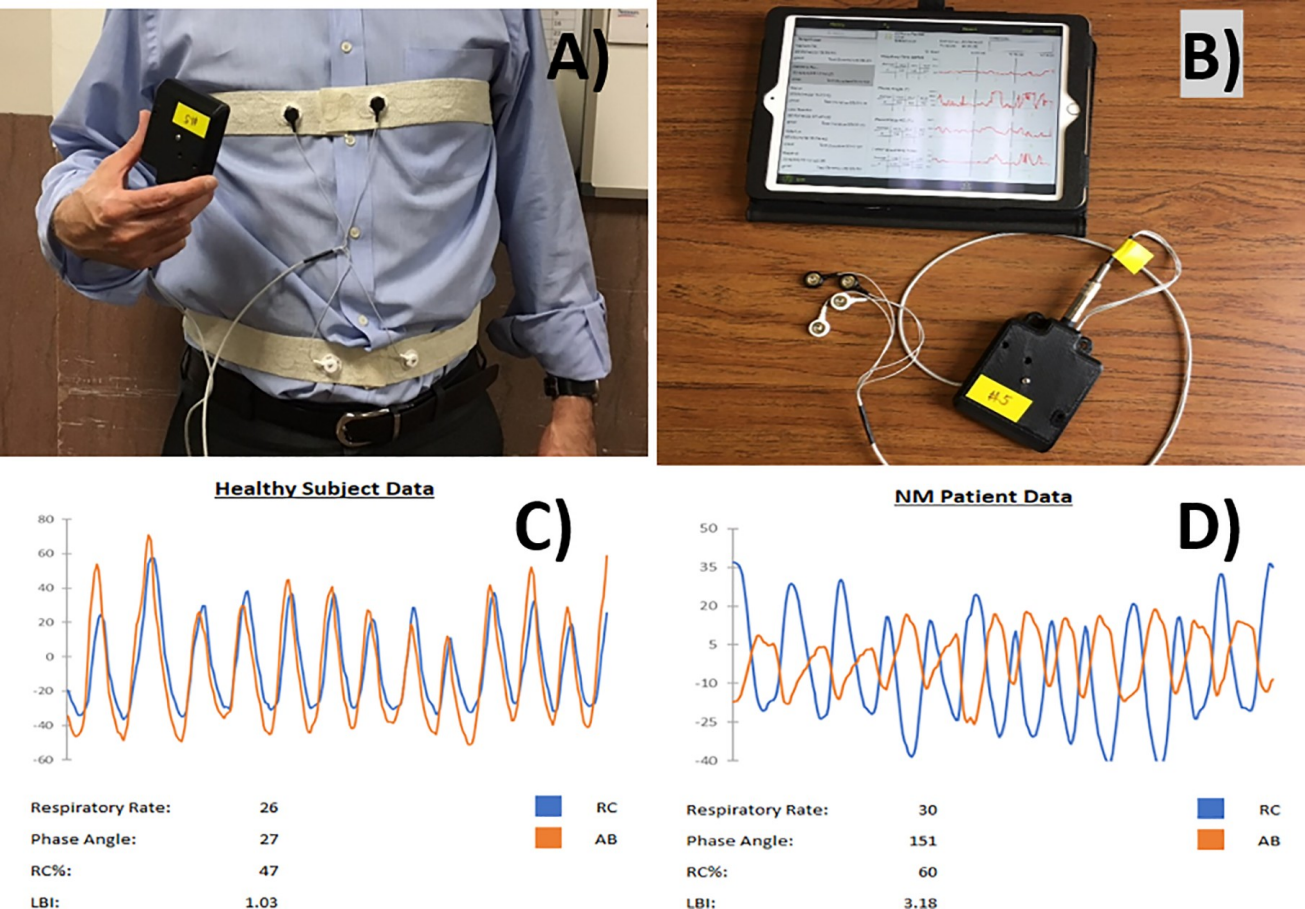
Example of *pneu*RIP ^TM^ operation, hardware, and data display. (A) The *pneu*RIP^TM^ device uses two bands: one band around the ribcage and one around the abdomen (illustrated by author (TR). (B) The *pneu*RIP^TM^ wirelessly connects to an iPad and displays data in real-time plots. (C) Typical RIP data from a healthy subject. (D) Typical data from a neuromuscular DMD patient (Image modified from Rahman et al [8]).

As shown in Fig 1, respiratory inductance plethysmography using the *pneu*RIP^TM^, (hardware and software provided by Creative Micro Designs, Newark, DE) was conducted on the 16 male participants (9 healthy subjects and 7 patients with DMD [ages 5–18]). The participants sat on a chair or medical table for 3–5 min while the device recorded the participant’s quiet breathing. The *pneu*RIP^TM^, wirelessly connected with an iPad (Apple, Cupertino, CA) to plot the data points and calculate the RIP parameters. All data were shown in real-time. After the tests, the data were saved securely for further analysis. Work of breathing (WOB) indices were recorded, which included labored-breathing index (LBI), phase angle (Φ) between abdomen and rib cage, respiratory rate (RR), and percentage of rib cage input (RC %), as previously described [8,11]. Heart rate (HR) was measured separately.

### RIP physiology and approach

Respiratory inductance plethysmography is a method that measures changes in cross-sectional area of the rib cage (RC) and the abdomen (ABD) [11]. The method consists of two elastic bands, each with an embedded insulated induction wire. Each band is placed around the RC and the ABD forming a coil (Fig 1A). Alternating current passes through the insulated wires and generates a self-inductance that oscillates in a cyclic pattern that tracks changes in the cross-sectional area of each respiratory compartment. Using *pneu*RIP^TM^,[8], a research device developed in collaboration with Creative Micro Designs, (Newark, DE) that provided the hardware transmitter and software (Fig 1B), these inductive signals are transmitted to an iPad and displayed (Fig 1C & 1D).

The signals recorded by the *pneu*RIP^TM^ from the elastic bands (Fig 1C & 1D) are mostly sinusoidal where each point is characterized by a magnitude and a phase. The magnitude represents the compartment change in volume and the phase angle defines the shift in RC and ABD signals. Typically, clinicians visually inspect the signals to see if the RC and ABD signals are synchronous. The *pneu*RIP^TM^ device records and displays the magnitude and phase of the signals and quantifies the absolute and relative movement of the RC and ABD through the extraction of WOB indices that are defined as follows:

1. Percent rib cage (RC%), which is a ratio of the absolute magnitude of RC signal to the sum of the absolute magnitudes of the RC and ABD signals [8,11]. The RC%, shown in equation (1), defines RC contribution to tidal volume and is presented as a percent of the combined RC and ABD volume changes.
  $$RC\%=\frac{\left|RC\right|}{\left|RC|+|ABD\right|}$$
  where |*RC*| is the magnitude of the signal recorded from the ribcage and |*ABD*| is the magnitude of the signal recorded from the abdominal compartment. The average RC% for a healthy subject (Fig 1C) is close to 50%, indicating that the RC and the ABD are contributing about the same amount to tidal volume. However, in Fig 1D, average RC% is 60%, indicating that RC is providing a greater contribution to tidal volume than ABD. The RC% in this case is outside the normal range (Age 5–18 yr] determined by Rahman et al [8] and Balasubramaniam et al [13] for sitting subjects.
2. Labored breathing index (LBI) (equation 2), which is a measure of respiratory effort due to asynchronous breathing [8, 11]. LBI is a ratio that is an estimate of WOB effort.
  $$LBI=\frac{{\left|RC\right|}_{t}^{inphase}+{\left|ABD\right|}_{t}^{inphase}}{{\left|RC\right|}_{t}+{\left|ABD\right|}_{t}}$$
  where |*RC*|_*t*_ and |*ABD*|_*t*_ are the magnitudes of the RC and ABD compartment signals at any instant of time (t), while ${\left|RC\right|}_{t}^{inphase}$ and ${\left|ABD\right|}_{t}^{inphase}$ are the magnitudes when the signals are in-phase. The denominator is the tidal volume (V_T_), which is the sum of the absolute values of the RC and ABD signals, as recorded. The numerator is the maximum compartmental volume and is the sum of the absolute values of the RC and ABD signals when in unison. When the RC and ABD signals are asynchronous as in Fig 1D, (LBI = 3.18) the LBI is larger than 1.0. In Fig 1C, since the signals are in-phase, the tidal volume and the maximum compartmental volume are almost the same and LBI = 1.03 which is close to 1.0 and normal [8,13].
3. Phase difference (ϕ) [8,11], which is a measure of synchrony between RC and ABD compartments. The phase angle (Φ) between the RC and ABD is calculated by normalizing the signals over 20 samples. The *ϕ* is then calculated by equation 3, expressed as follows:
  $$\phi =\cos^{-1}\frac{\sum_{n=0}^{N-1}x(n)y(n)}{\sqrt{\left[\sum_{n=0}^{N-1}{x(n)}^{2}\sum_{n=0}^{N-1}{y(n)}^{2}\right]}}$$
  where x represents RC,y represents ABD, and n is the number of samples.
  When the signals from the RC and ABD are synchronized as seen in (Fig 1C), the difference in phase (ϕ = 27 degrees) is in the normal range of 0 < ϕ < 30 degrees [8,13]. However, the phase difference increases when the two signals become asynchronous as shown in (Fig 1D). The average phase difference reported for the two plots in Fig 1D indicates asynchrony, since ϕ = 151 degrees, which is outside the normal range.
4. Respiratory rate in breaths per minute. This was determined by a fast Fourier transform (FFT) algorithm to generate the average magnitudes for each frequency of the RC and ABD signal. From these calculations, an array is generated from which the largest value is selected, as well as amplitudes within 20% of this value. Finally, we compute a weighted average using indices of these values. This value is the frequency multiplied by 60 to obtain breaths per minute.

The raw data for the Φ, RC%, RR, and LBI of each participant were displayed in histogram plots to identify distribution patterns within the normal ranges [8,13]. The percentages of data values within the normal ranges were compared between healthy volunteers and DMD patients.

### Data analysis

All 16 participants were males aged 5–18 years, studied for up to 5 min. with RIP measurements every 0.1 sec. Quantitative WOB indices over time were aggregated for each subject. Summarized data were presented in tabular form as mean and standard error (SEM). A two-sample (unpaired) t- test was performed to compare mean aggregated WOB indices between the healthy and DMD groups. Box plots, as well as Shapiro-Wilk tests, of aggregated WOB indices were performed, and no deviation from normality was observed.

The distribution of WOB index measurements within their respective normal ranges [13] was summarized for each subject as percentages. A two-sample t-test was performed to compare the mean percent differences in WOB index measurements within the normal range between healthy and DMD patients. Based on the recent review of normative RIP indices [13] for sitting subjects [Ages 5–18] and our previous study [8], we established tight criteria for the normal ranges: mean Φ (0 ≤ Φ ≤ 30) degrees; median RC% ± 10%; median RR ± 5%, and mean LBI (1 ≤ LBI ≤ 1.1). In addition, histograms of each participant’s Φ, RC%, RR, and LBI were plotted to compare the shape of the data distribution between study groups. All tests were two tailed with a level of significance of *P* < 0.05. Statistical software SPSS version 23 (IBM, Armonk, NY) and Excel 2016 (Microsoft, Redmond, WA) were used to perform the statistical analysis and data plotting.

## Results

With regard to the feasibility of noninvasive pulmonary testing, it is noteworthy that all subjects were compliant with performing RIP studies using *pneu*RIP^TM^ (100% compliance). The participants in this study were separated into two groups: 7 DMD patients and 9 healthy children. The two-sample (unpaired) t-test results comparing mean and SEM of aggregated WOB indices between the healthy and DMD groups are shown in Table 2. The variables include the WOB indices (Ф, RC %, RR, LBI) and HR. As shown, DMD patients had a statistically significant elevation in Φ, LBI, and HR averages (*P* < 0.006, *P* < 0.002, *P* < 0.046, respectively). RR and RC% means in healthy subjects and DMD patients were not different (for complete data, see S1 Table).

**Table 2:** Data analysis of mean and SEM of WOB indices and heart rate.

Factors	Phase Angle (degrees)		Respiratory Rate (Br/min)		% Ribcage Contribution		Labored Breathing Index		Heart Rate (B/min)	
	Mean	SEM	Mean	SEM	Mean	SEM	Mean	SEM	Mean	SEM
**Healthy**	23.40	4.33	11.75	1.22	56.07	2.57	1.07	0.02	80.56	2.70
**Patient**	60.18	10.41	11.39	0.88	46.28	4.21	1.23	0.04	94.14	5.24
***P* Value**	0.006		0.818		0.074		0.002		0.046	

**Fig 2:**
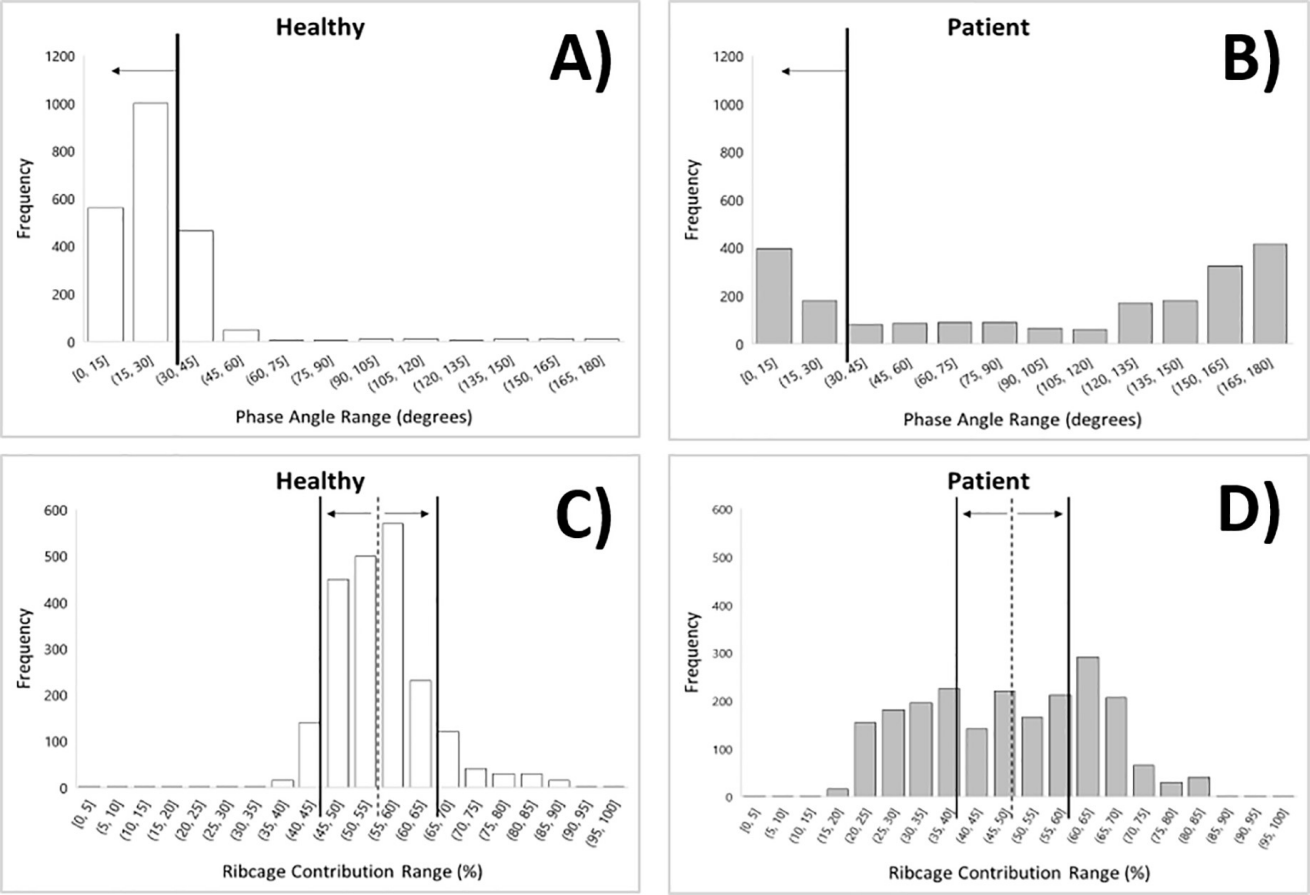
Histograms showing differences in phase angle and percent ribcage contribution: healthy subject vs. patient with DMD. (A) Phase angle histogram of a healthy subject; 71.7% of the data values are within the normal range (0 ≤ φ ≤ 30) degrees. (B) Phase angle histogram of a DMD patient; only 27.0% of the data values are within the normal range (0 ≤ φ ≤ 30) degrees. (C) Percent of ribcage contribution histogram of a healthy subject; 83.1% of the data values are within the normal range with median being 55% (45% ≤ RC% ≤ 65%). (D) Percent of ribcage contribution histogram of a DMD patient; 37.0% of the data values are within the normal range with median being 49%.

After the initial analysis, data from each participant were plotted into histograms, one each for Φ, RR, RC %, and LBI. Once plotted, the Φ histograms showed two clear patterns. The first pattern, seen mostly in the healthy participants’ histograms, depicted a single clustering of data, typically below 30 degrees. The second pattern, seen mostly in the DMD patients’ histograms, is more diffuse, with bi-modal separation. The second pattern displays the data dispersing far beyond the typical range of normal data. It was determined that a similarly diffuse distribution pattern was emerging on all the parameter histograms for the DMD patients. As presented in the data analysis section, the normal ranges [8,13] of each variable were used to compare and contrast distribution patterns. Typical histogram plots for Φ and RC% are shown in Fig 2 along with the normal range [8,13]. The plot of the healthy subject clearly shows a clustering of measured data points within the normal range while the patient’s data values are more variable. As shown in Fig 2, the phase angle histogram of a healthy subject demonstrated that 71.7% of the phase data values are within the normal range (0 ≤ φ ≤ 30 degrees), whereas only 27.0% of the phase data values for the DMD patient are within the normal range [8,13]. Also shown, the RC% histogram of a healthy subject demonstrated that 83.1% of the data values are within the normal range (45% ≤ RC% ≤ 65%) with a median of 55%; in contrast, only 37.0% of the RC% data values for the DMD patient are within the normal range.

For additional evaluation, the percentage of RIP data values within the normal range for each participant was calculated and averaged for comparison (Table 3). Unpaired t-tests determined significance of RIP data between each study group. All DMD data distributions were statistically different from healthy subjects based on the analysis of histograms. While the healthy subjects had an average of 82.06% Φ, 88.71% RR, 73.81% RC%, and 87.30% LBI of the RIP data within normal ranges, the DMD patients showed significantly less RIP data within the normal ranges, with only 49.7% Φ (*P* < 0.004); 48.0% RC% (*P* < 0.008); 69.2% RR (*P* < 0.044), and 50.7% LBI (*P* < 0.002).

**Table 3:** Histogram data of mean (SEM) and median (+/- percentile) WOB indices expressed as percent within the normal range*.

Factors	Phase Angle Mean 0≤Φ≤30 degrees	Respiratory Rate Median RR±5%	% Ribcage Contribution Median RC%±10%	Labored Breathing Index Mean 1≤LBI≤1.1
**Healthy**	82.06% (5.12%)	88.71% (5.17%)	73.81% (6.78%)	87.30% (4.42%)
**Patient**	49.71% (8.28%)	69.22% (6.94%)	48.02% (6.55%)	50.68% (8.21%)
***P* Value**	0.004	0.044	0.008	0.002

*Normal Ranges based on data from Rahman et al [8] and Balasubramaniam et al [13]

## Discussion

Due to the degenerative nature of DMD, patients require constant monitoring of symptoms starting from young ages. Many pulmonary function tests are demanding and rely on patient cooperation, so these tests are not applicable for those patients with DMD who cannot cooperate with standard pulmonary testing due to their age or disability. In contrast, pneuRIP^TM^ technology offers an effortless, noninvasive pulmonary assessment that can be completed during outpatient visits, or possibly within the home, with minimal patient cooperation or discomfort as demonstrated in multiple neonatal studies [10–12].

This study analyzed DMD patients’ data against age-matched healthy control data, as well as established age-matched data from previously reported studies [8,13], with a primary outcome that *pneu*RIP^TM^ can demonstrate differential WOB indices and breathing patterns between these groups of children in a hospital clinic setting. Also of note, all patient studies were completed within the regularly scheduled clinic visit. In addition, there was 100% compliance with subjects completing testing.

As shown above in tabular and graphic format, the initial analysis found that DMD patients displayed elevated means in Φ, LBI, and HR compared to healthy age-matched controls (Table 2). In a recent emergency department study, Giordano et al [14] reported that Ф, LBI, and HR were all elevated in patients who were admitted to the hospital for asthmatic exacerbations as compared with those discharged. One could speculate that the increase in HR may be associated with an increase in cardiac output due to the elevation in phase angle and labored breathing in both studies.

Further analysis of individual histograms of each participant’s RIP data showed two clear patterns in the distribution of samples: a distinct clustering or a diffused spread. Analysis of the percentage of RIP values within the normal ranges [8,13] demonstrated a clear disparity between healthy subjects’ tight, single-distribution clustering and DMD patients’ more diffused, even bi-modal, distributions. Although this is the first RIP pattern distribution study involving children with DMD, a previous study involving pre-term infants on noninvasive respiratory support (spontaneous breathing) noted a similar diffuse, bi-modal pattern in phase-angle data distribution [10]. The infant study showed that WOB indices in newborns with respiratory insufficiency receiving high-flow nasal cannula and nasal continuous positive airway pressure demonstrated a similar Φ data pattern (bi-modal clustering). Although the disease process and ages are clearly different, we also observed this distribution pattern in our patients with DMD. However, it is noteworthy that both preterm infants and patients with DMD have compromised restrictive lung problems and weak respiratory muscles. On this basis, we speculate that patients with DMD may use a similar strategy for conserving muscle effort and minimizing respiratory muscle fatigue. Thus, rather than fatiguing both muscle groups with continuous synchronous breathing, both preterm infants and DMD patients may be sparing muscle efforts by switching between rib cage and abdominal contributions to tidal breathing.

Gauld et al utilized forced oscillation techniques (FOT) in 12 children (<10 yrs) with spinal muscular atrophy, over a 3-month period. Although they reported spirometry and peak cough flow measurements were possible, there was only 30% compliance for spirometry [15]. This study demonstrates the difficulty some neuromuscular patients have with more demanding pulmonary function assessments. In addition, they found that the FOT methods detected abnormal levels of resistance and reactance in SMA patients that worsened over a 12-month period; there was no correlation with clinical characteristics.

Regarding the severity of respiratory involvement, we and others have previously demonstrated the utility of RIP measurements in identifying abnormal WOB indices and patterns of breathing in neuromuscular disorders [9], in preterm infants with RDS [10], in BPD patients in the NICU [11], and in patients who were admitted to the hospital for asthmatic exacerbations [14]. As noted herein, infant RDS, bronchopulmonary dysplasia, and asthmatic patient studies demonstrate that RIP technology can differentiate muscular from non-muscular causes of respiratory insufficiency.

Our study had a few inherent limitations. Since the study was a pilot study to determine whether *pneu*RIP^TM^ can demonstrate differential WOB indices and breathing patterns in children with DMD as compared to age-matched healthy subjects in a hospital clinic setting, the initial results are based on alimited sample size. Therefore, our study results need to be interpreted with caution in that our DMD study group may not represent a larger DMD population with respect to key developmental changes in pulmonary function. We emphasize that our intention was not to look at the progression of RIP data in the DMD population at this time. However, it is anticipated that these positive outcomes, with regard to feasibility, test compliance (100% in all consented subjects), and potential predictability of asynchronous breathing and diffuse breathing patterns will enable us to perform a much larger prospective study in which *pneu*RIP^TM^ data will be utilized in patient disposition and therapy guidance. Post-hoc analysis of the enrolled patients, again due to sample size, did not allow a uniform distribution of patients with regard to age, race, or severity of disease.

In conclusion, in this study we demonstrated the utility of noninvasive *pneu*RIP^TM^ techniques to measure WOB indices and breathing patterns in children with DMD against age-matched healthy controls. As reported, patients with DMD showed abnormal results with increased markers of TAA, WOB indices, and diffuse breathing patterns likely resulting from neuromuscular weakness. In addition, it is noteworthy that there was 100% patient compliance using *pneu*RIP^TM^ instrumentation, demonstrating diagnostic feasibility in a clinical setting and possibly other hospital settings. The evaluation of TAA breathing pattern distributions in addition to compromised WOB indices (differences in Φ, LBI, and HR) using RIP technology may provide valuable insight in the progression of DMD in children. As noted at the outset, the *pneu*RIP^TM^ testing approach may provide useful information in both inpatient and outpatient settings, and possibly in-home settings, since information can be transmitted securely to the hospital medical records. Furthermore, from a clinical perspective, the new mutation- and gene-specific therapies for NM diseases, like DMD, are likely to confer long-term therapeutic benefit in the future. Thus, appropriate, noninvasive approaches to assess therapeutic outcomes in respiratory function will be required in children with DMD—particularly the youngest patients who cannot perform standard pulmonary testing. Finally, further studies should compare both the results from the *pneu*RIP^TM^ to those of other pulmonary function tests as well as the results from patients with DMD to those who have other neuromuscular diseases.

## Supporting information

S1 Table Respiratory inductance plethysmography distribution and mean values for healthy subjects and DMD patients. (XLSX)

## References

[1] Khirani S, Ramirez A, Aubertin G, Boulé M, Chemouny C, Forin V, et al Respiratory muscle decline in Duchenne muscular dystrophy. Pediatr Pulmonol. 2014;49: 473–481. 10.1002/ppul.22847 23836708

[2] LoMauro A, D'Angelo MG, Aliverti A. Assessment and management of respiratory function in patients with Duchenne muscular dystrophy: current and emerging options. Ther Clin Risk Manag. 2015;11: 1475–1488. 26451113 10.2147/TCRM.S55889 PMC4592047

[3] Saito W, Mizuno K, Inoue G, Imura T, Nakazawa T, Miyagi M, et al Perioperative Evaluation of Respiratory Muscle Strength after Scoliosis Correction in Patients with Duchenne Muscular Dystrophy. Asian Spine J. 2017;11: 787–792. 10.4184/asj.2017.11.5.787 29093790 PMC5662863

[4] Daftary AS, Crisanti M, Kalra M, Wong B, Amin R. Effect of long-term steroids on cough efficiency and respiratory muscle strength in patients with Duchenne muscular dystrophy. Pediatrics. 2007;119: e320–e324. 10.1542/peds.2006-1400 17272595

[5] Victor RG, Sweeney HL, Finkel R, McDonald CM, Byrne B, Eagle M, et al A phase 3 randomized placebo-controlled trial of tadalafil for Duchenne muscular dystrophy. Neurology. 2017;89: 1811–1820. 10.1212/WNL.0000000000004570 28972192 PMC5664308

[6] Fauroux B, Khirani S. Neuromuscular disease and respiratory physiology in children: putting lung function into perspective. Respirology. 2014;19: 782–791. 10.1111/resp.12330 24975704

[7] Steier J, Kaul S, Seymour J, Jolley C, Rafferty G, Man W, et al The value of multiple tests of respiratory muscle strength. Thorax. 2007;62: 975–980. 10.1136/thx.2006.072884 17557772 PMC2117126

[8] Rahman T, Page R, Page C, Bonnefoy JR, Cox T, Shaffer TH. pneuRIP^TM^: A Novel Respiratory Inductance Plethysmography Monitor. J Med Device. 2017;11: 0110101–110106. 10.1115/1.4035546 28289485 PMC5318692

[9] Perez A, Mulot R, Vardon G, Barois A, Gallego J. Thoracoabdominal pattern of breathing in neuromuscular disorders. Chest. 1996;110: 454–461. 10.1378/chest.110.2.454 8697851

[10] de Jongh BE, Locke R, Mackley A, Emberger J, Bostick D, Stefano J, et al Work of breathing indices in infants with respiratory insufficiency receiving high-flow nasal cannula and nasal continuous positive airway pressure. J Perinatol. 2014;34: 27–32. 10.1038/jp.2013.120 24071905 PMC4141486

[11] Allen JL, Greenspan JS, Deoras KS, Keklikian E, Wolfson MR, Shaffer TH. Interaction between chest wall motion and lung mechanics in normal infants and infants with bronchopulmonary dysplasia. Pediatr Pulmonol. 1991;11: 37–43. 10.1002/ppul.1950110107 1833720

[12] Wolfson MR, Greenspan JS, Deoras KS, Allen JL, Shaffer TH. Effect of position on the mechanical interaction between the ribcage and abdomen in preterm infants. J Appl Physiol. 1992;72: 1032–1038. 10.1152/jappl.1992.72.3.1032 1533209

[13] Balasubramaniam SL, Wang Y, Ryan L, Hossain J, Rahman T, Shaffer TH. Review Article: Age-related ranges of respiratory inductance plethysmography (RIP) reference values for infants and children. Paediatric Respiratory Reviews. (2018), 10.1016/j.prrv.2018.03.010 30799137

[14] Giordano K, Rodriguez E, Green N, et al Pulmonary function tests in emergency department pediatric patients with acute wheezing/asthma exacerbation. Pulm Med. 2012;2012:724139 10.1155/2012/724139 23304496 PMC3523566

[15] Gauld LM, Keeling LA, Shackleton CE, Sly PD. Forced oscillation technique in spinal muscular atrophy. Chest. 2014;146: 795–803. 10.1378/chest.14-0166 24810887

